# Biosynthesis of plant neuroactive alkaloids treating Alzheimer’s disease

**DOI:** 10.3389/fphar.2025.1500955

**Published:** 2025-02-05

**Authors:** Quanyu Yin, Zhengkang Zhu, Mengquan Yang

**Affiliations:** National Tobacco Cultivation, Physiology and Biochemistry Research Center, Flavors and Fragrance Engineering and Technology Research Center of Henan Province, College of Tobacco Science, Henan Agricultural University, Zhengzhou, Henan, China

**Keywords:** Alzheimer’s disease, biosynthesis, alkaloid, huperzine A, galantamine

## Introduction

Alzheimer’s disease (AD) is a debilitating neurodegenerative disorder characterized by cognitive decline and memory impairment. With increasing global prevalence, the need for effective therapeutic interventions is critical. Among the currently approved treatments, acetylcholinesterase inhibitors (AChEIs) like huperzine A and galantamine stand out due to their neuroprotective roles. These plant-derived alkaloids have demonstrated significant efficacy in alleviating symptoms by increasing acetylcholine levels in the brain.

While numerous other plant alkaloids exhibit varying degrees of neuroactive properties, huperzine A and galantamine remain the only plant-derived alkaloids currently approved and marketed as specific treatments for AD and other neurodegenerative diseases. For example, alkaloids such as berberine (from *Berberis* species) and rhynchophylline (from *Uncaria rhynchophylla*) have shown potential in targeting amyloid-beta (Aβ) aggregation, oxidative stress, and tau hyperphosphorylation. Similarly, harmine has demonstrated the ability to inhibit tau hyperphosphorylation through dual inhibition of glycogen synthase kinase-3 beta (GSK-3β) and dual specificity tyrosine phosphorylation regulated kinase 1A (DYRK1A). However, these metabolites have not yet advanced to clinical applications (Ng et al., 2015; Rezaul Islam et al., 2024).

The elucidation of the biosynthetic pathways of huperzine A and galantamine marks a significant advancement in understanding plant biochemistry and specialized metabolism. It not only advances our understanding of plant-derived neuroactive metabolites but also provides opportunities for sustainable and scalable production through synthetic biology approaches. By leveraging this approach, researchers can reconstruct the biosynthetic pathways of plant-derived natural products in microbial or plant systems, facilitating efficient production and reducing the reliance on native plant sources for these valuable compounds (Liu et al., 2023; Zhang et al., 2023; Bai et al., 2024; Teng et al., 2024). This opinion highlights the implications of these discoveries for future research and application in neurodegenerative disease treatment.

### Huperzine A: a lycopodium alkaloid

**FIGURE 1:**
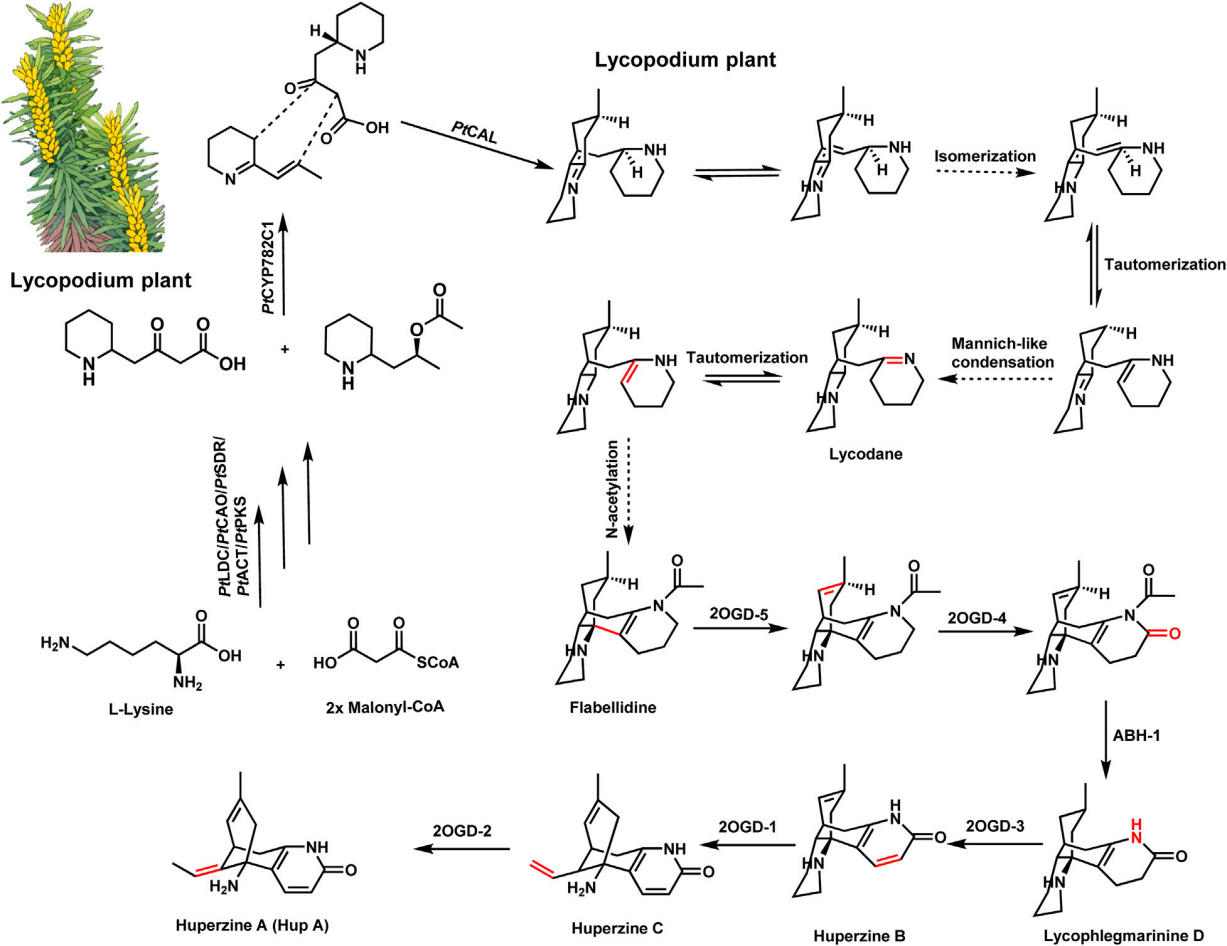
Schematic illustration of the biosynthesis of lycopodium alkaloids. Biosynthetic pathway of huperzine A (HupA). PtLDC, lycine decarboxylase; PtCAO, copper amine oxidase; PtPKS, piperidyl ketide synthase; PtSDR, short-chain dehydrogenase/reductase; PtCAT, acetyltransferase; PtCAL, alpha-carbonic anhydrases-like; 2OGD-1, 2-oxoglutarate-dependent dioxygenase 1; 2OGD-2, 2-oxoglutarate-dependent dioxygenase 2; 2OGD-3, 2-oxoglutarate-dependent dioxygenase 3; 2OGD-4, 2-oxoglutarate-dependent dioxygenase 4; 2OGD-5, 2-oxoglutarate-dependent dioxygenase 5.

Huperzine A, derived from *Huperzia serrata* (Lycopodiaceae), is a well-known AChEI that has been widely used in traditional Chinese medicine (Ma and Gang, 2004; Yang et al., 2017; Wang et al., 2020; Zhang et al., 2024). The elucidation of the biosynthetic pathway of huperzine A has provided crucial insights into the formation of Lycopodium alkaloids and uncovered numerous enzymes with novel functions (Li et al., 2022; Ushimaru and Abe, 2023; Cheng et al., 2024). Recent studies have identified three novel neofunctionalized α-carbonic anhydrase-like (CAL) enzymes responsible for the key Mannich-like condensations that form core carbon–carbon bonds in Lycopodium alkaloids, key steps in the construction of their polycyclic skeletons. Through transcriptome analysis and enzyme characterization, Nett et al. identified key enzymes such as CAL-1 and CAL-2, which promote crucial annulation reactions (Nett et al., 2023; Liu F. et al., 2024; Zamar et al., 2024). The pathway proceeds through stereospecific modifications and scaffold tailoring, involving additional enzymes like Fe(II)-dependent dioxygenases, which introduce oxidation steps crucial for the final bioactive form of huperzine A (Figure 1) (Nett et al., 2021; Nett et al., 2023; Ushimaru and Abe, 2023). These findings shed light on the complex evolution of neuroactive alkaloids in Lycopodium species, suggesting that such enzymes have evolved for specialized metabolite production as a defense mechanism.

Moreover, transient expression of huperzine A biosynthetic genes in *Nicotiana benthamiana* allowed for the successful production of Lycopodium alkaloid congeners, underscoring the potential for scalable biosynthesis through heterologous platforms. This breakthrough not only deepens our understanding of plant-derived alkaloids but also opens the door to bioengineering huperzine A production in microbial or plant chassis, reducing reliance on natural resources (Zhang et al., 2022; Gao et al., 2023; Liu et al., 2023; Bai et al., 2024; Golubova et al., 2024).

### Galantamine: an amaryllidaceae alkaloid

**FIGURE 2:**
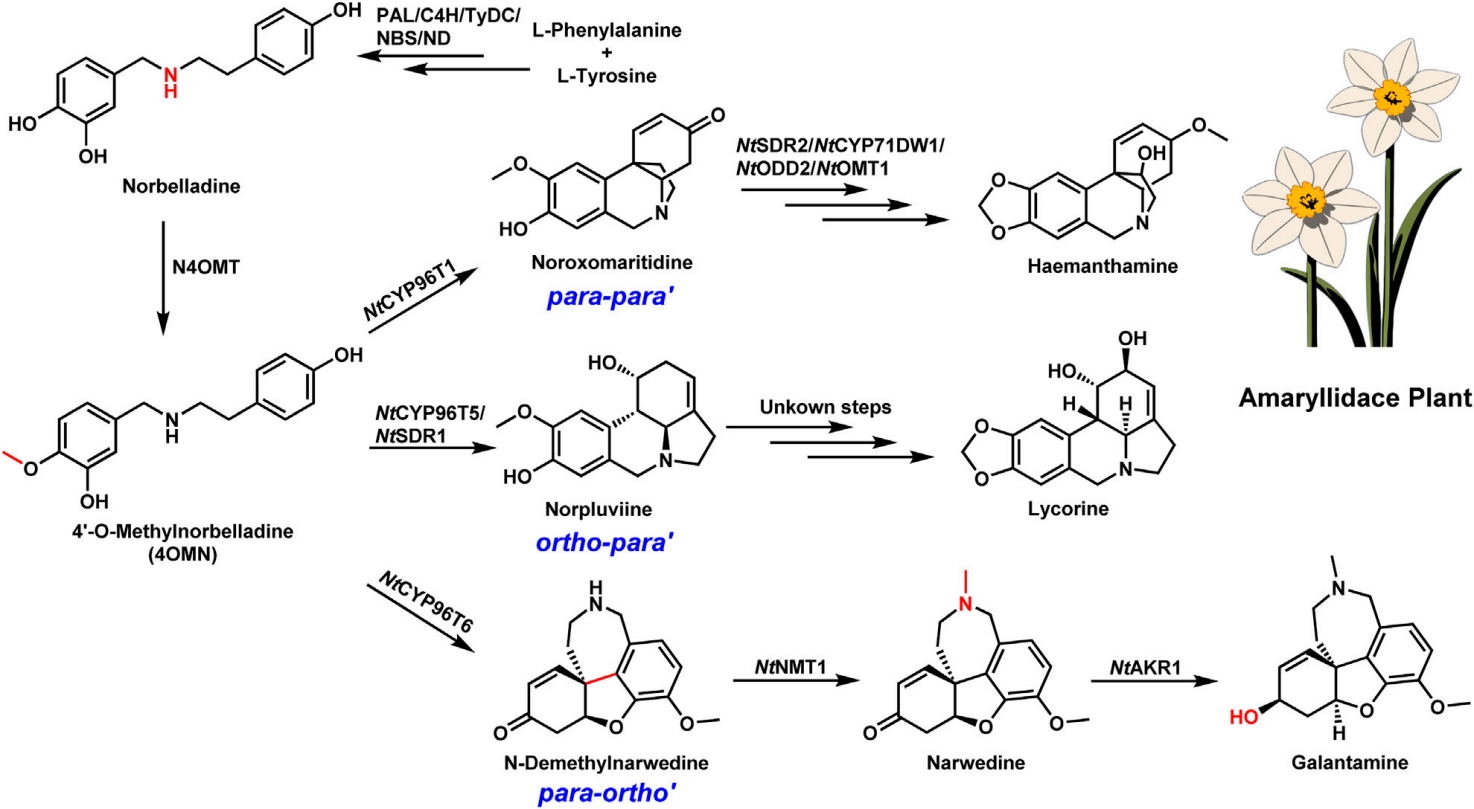
Schematic illustration of the biosynthesis of amaryllidaceae alkaloids. Biosynthetic pathway of galantamine. PAL, phenylalanine ammonia-lyase; C4H, cinnamate 4-hydroxylase; TyDC, tyrosine decarboxylase; NBS, norbelladine synthase; ND, norcraugsodine reductase; NtSDR2, short-chain dehydrogenase/reductase 2; NtCYP71DW1, cytochrome P450 71DW1; NtODD2, 2-oxoglutarate-dependent dioxygenase 2; NtOMT1, O-Methyltransferase 1; NtCYP 96T1, cytochrome P450 96T1; NtCYP 96T5, cytochrome P450 96T5; NtCYP 96T6, cytochrome P450 96T6; NtSDR1, short-chain dehydrogenase/reductase 1; NtNMT1, N-demethylnarwedine methyltransferase 1; NtAKR1, aldo-keto reductase 1. Dashed arrows represent steps that are hypothesized to occur spontaneously or without enzymatic catalysis.

Galantamine, an alkaloid derived from plants in the *Amaryllidaceae* family, particularly daffodils (*Narcissus* spp.), is another crucial AChEI used in AD treatment (Prvulovic et al., 2010). Similar to huperzine A, the biosynthetic pathway of galantamine was recently elucidated, providing invaluable insights into its production (Kilgore et al., 2014; Li et al., 2018; Li et al., 2019; Hu et al., 2021; Mehta et al., 2024). The discovery began with identifying the key precursor, 4′-O-methylnorbelladine (4OMN), followed by oxidative coupling catalyzed by cytochrome P450 enzymes such as NtCYP96T6. This enzyme facilitates the para-ortho (p-o’) oxidative coupling necessary to produce the galantamine skeleton. Subsequent methylation and reduction steps, catalyzed by NtNMT1 and NtAKR1 respectively, complete the biosynthesis of galantamine (Figure 2) (Mehta et al., 2024).

This discovery has profound implications for synthetic biology and metabolic engineering. With galantamine currently sourced primarily from natural populations of daffodils, the ability to biosynthesize it through engineered microbial systems holds significant promise for sustainable and scalable production (Zhang et al., 2022; Gao et al., 2023). Additionally, the elucidation of galantamine’s pathway helps to understand how plants generate chemical diversity from simple precursors, providing a foundation for engineering other related alkaloids with potential therapeutic value.

## Challenges and future directions

The elucidation of huperzine A and galantamine biosynthetic pathways underscores the complexity and elegance of plant specialized metabolism. Both alkaloids share the common feature of acting as acetylcholinesterase inhibitors, though their evolutionary and biosynthetic origins differ significantly. The Lycopodium and Amaryllidaceae families, through distinct evolutionary pressures, have developed highly specialized enzymes that allow these plants to synthesize neuroactive metabolites with intricate polycyclic structures. While the elucidation of these biosynthetic pathways represents a significant advancement, several challenges remain.

First, the *in vivo* functional roles of these alkaloids in plants are not fully understood. It is speculated that they serve as defense metabolites against herbivores, but the regulatory mechanisms governing their production remain elusive (Chavez et al., 2024). Further research into the ecological roles of these alkaloids could provide important insights into the evolution of medicinal plants, the evolution of biosynthetic pathways, and their interactions with the environment (Szypuła and Pietrosiuk, 2023; Zhang et al., 2024).

Second, the scalability of producing huperzine A and galantamine through heterologous systems remains a key challenge. While transient expression in *N. benthamiana* has demonstrated proof-of-concept for biosynthesis, translating these findings into industrial-scale production will require optimization of gene expression, precursor supply, and enzymatic activity in microbial or plant-based platforms (Liu J. C. et al., 2024; Yang et al., 2024). Optimizing precursor supply, enhancing enzyme activity, and achieving high-yield production in heterologous systems are critical bottlenecks. Microbial synthetic biology platforms, such as *Saccharomyces cerevisiae* and *Pichia pastoris*, offer promising avenues for large-scale production due to their scalability and ease of genetic manipulation (Zhang et al., 2022; Gao et al., 2023; Yang et al., 2024). On the other hand, plant chassis like *N. benthamiana* provide unique advantages, including natural metabolic environments and compartmentalized cells conducive to complex biosynthesis (Liu et al., 2023; Zhang et al., 2023; Golubova et al., 2024; Liu J. C. et al., 2024). Advances in CRISPR-based genome editing, multi-gene pathway assembly, and metabolic flux optimization are pivotal for overcoming current limitations (Liao et al., 2023; Xie et al., 2023; Teng et al., 2024). By leveraging these tools, researchers can create efficient production platforms not only for huperzine A and galantamine but also for other plant-derived neuroactive alkaloids, paving the way for accessible and sustainable therapeutics for Alzheimer’s disease.

Finally, the potential for discovering new neuroactive alkaloids in related plant species should not be overlooked. The pathways for huperzine A and galantamine likely represent only a fraction of the neuroactive metabolites that plants produce. Systematic exploration of the metabolic pathways in related species could yield novel AChE inhibitors or other metabolites targeting neurodegenerative diseases.

## Conclusion

The elucidation of the biosynthetic pathways of huperzine A and galantamine marks a pivotal moment in plant biochemistry and neuropharmacology. These discoveries not only deepen our understanding of plant metabolism but also offer practical pathways for the sustainable production of crucial AD treatments. As the global population ages and the burden of neurodegenerative diseases grows, plant-derived neuroactive alkaloids like huperzine A and galantamine will continue to play an essential role in treatment. The future of this research lies in the intersection of synthetic biology, metabolic engineering, and traditional plant sciences, paving the way for innovative solutions to Alzheimer’s disease and other neurological disorders.
